# The factor structure and construct validity of the parent-reported Inventory of Callous-Unemotional Traits among school-aged children and adolescents

**DOI:** 10.1371/journal.pone.0221046

**Published:** 2019-08-16

**Authors:** Satomi Yoshida, Masaki Adachi, Michio Takahashi, Nobuya Takanyanagi, Sayura Yasuda, Hirokazu Osada, Kazuhiko Nakamura

**Affiliations:** 1 Department of Neuropsychiatry, Graduate School of Medicine, Hirosaki University, Hirosaki, Aomori, Japan; 2 Research Center for Child Mental Development, Graduate School of Medicine, Hirosaki University, Aomori, Japan; 3 Faculty of Human Studies, Aichi Toho University, Nagoya, Aichi, Japan; 4 Department of Psychology, School of Human Sciences, Senshu University, Tokyo, Japan; Chiba Daigaku, JAPAN

## Abstract

In this study, we assessed the factor structure and construct validity of the parent-reported Inventory of Callous-Unemotional Traits (ICU) among school-aged children and adolescents, aged 6 to 15 years, in a community setting in Japan (n = 10,936). We investigated 15 models that have been reported in previous studies and used confirmatory factor analyses to determine a model that might actually be the best-fit among these. We then examined the correlations between the score of ICU and the Strengths and Difficulties Questionnaire (SDQ) in the best fit model and the three-factor bifactor (3FBF) model with the original ICU through cross-sectional and longitudinal analysis to determine the concurrent and predictive validity of the ICU. The results showed that the best-fit model was the two-factor bifactor (2FBF) model with a revised version of the ICU with 12 items, excluding all but one item of unemotional factors. The cross-sectional and longitudinal analysis showed that higher general callous-unemotional factor scores, callousness and uncaring specific factor scores were significantly associated with a higher level of conduct problems and a lower level of prosocial behaviors in the SDQ. These tendencies were shown both in the 2FBF model with the revised version of the ICU and the 3FBF model with the original ICU. We conclude that the 2FBF model was useful for school-aged community samples, as it predicts increases in conduct problems and decreases in prosocial behavior with fewer items than the 3FBF model.

## Introduction

Conduct Disorder (CD) is highly heterogeneous in terms of severity, course, and etiology [1], and its incidence has been associated with criminal behavior and social exclusion, with an attendant range of costs to impacted individuals and society as a whole [2,3]. The variability of CD manifestations has contributed to the difficulties in its diagnosis and treatment. Therefore, clarifying the diagnostic ambiguities is significant from both research and clinical perspectives.

Over the past two decades, the concept of callous and unemotional traits (CU traits) has been regarded as a critical construct used to distinguish severe CD, and it generally supports a better understanding of the disorder. The theoretical framework of CU traits was initially derived from the concept of psychopathy in adults [4], and the condition is characterized by specific emotional reactions such as the absence of guilt and constricted displays of emotion, as well as elements of interpersonal style such as a failure to show empathy and the use of others to advance personal interests [5]. Previous studies have demonstrated the predictive utility of CU traits, and results have suggested that early detection of CU traits might help the implementation of early treatment for young people who have a high risk of severe CD, thus possibly lessening the severity of its behavioral impacts [6,7] at the individual and societal level.

In accordance with the expanding research demonstrating the utility of assessing CU traits, CU traits have recently been included in the Diagnostic and Statistical Manual of Mental Disorders, 5th edition (DSM-5) [8] as the specifier of CD. The DSM-5 delineates limited prosocial emotions as a subcategory of CD, and CU traits is one of the characteristics of limited prosocial emotions [9,10]. As the concept of CU traits has been applied to wider areas from clinical work to research, the importance of developing a comprehensive and reliable measure of CU traits has become increasingly apparent. However, many gaps remain in the underlying construct of CU traits, and it is essential to address these limitations to develop a more reliable measure.

The Inventory of Callous-Unemotional Traits (ICU) is one of the most widely used measurements to assess CU traits [11]. It was developed based on the Antisocial Process Screening Device, which screens a range of psychopathological dimensions, including CU traits, narcissism, and impulsivity in youth [12]. Among such characteristics, CU traits have consistently been identified as a distinct dimension among clinical and non-clinical children and adolescents [13,14], and the ICU was specified to assess the trait using three subscales, namely callousness, uncaringness, and lack of emotionality. There have been some validation studies that reported the ICU as a promising measurement to examine CU-traits. While initial studies showed the concurrent validity of the ICU [15–18], its construct validity [19] and predictive validity [20, 21] have also been reported recently. In terms of validation study of the ICU, some studies have employed the Strengths and Difficulties Questionnaire (SDQ) to investigate validity of the ICU. For example, Viding et al. [22] identified a significant positive correlation with CU traits measured by the ICU, and conduct problem and significant negative correlation with prosocial behaviours in the SDQ. Those relationships were also reported in cross-sectional [23] and longitudinal [24] studies.

The ICU is used for investigating CU traits among adolescents and young adults, and it has recently been introduced for use among children under ten years old [25,26]. A parent-reported ICU aids in the assessment of young children who are difficult to assess via self-evaluation questionnaires. However, its factor structure has not yet been well established, and its validity and reliability still need confirmation. To our knowledge, ten studies have investigated the factor structure of parent-reported ICU. The oldest among these investigations identified the best-fit as the three-factor bifactor (3FBF) model, which includes a general callous-unemotional (CU) factor along with the three specific factors of callousness, uncaring, and unemotional [27]. Although this model was widely used in subsequent research [28–30], other studies have demonstrated that the model does not satisfy sufficient model-fit criteria and suggested alternative models showing better fit qualities [31–34]. However, few studies have investigated the reproducibility of newly suggested factor structures.

A discussion about factor structures of the parent-reported ICU, as suggested in the previous research, can be summarized into three points: the number of factors, the hierarchy of factors, and the number of items included in the scale. First, the suggested number of factors ranges from two to three, with varying content [32–34]. Three-factor (3F) models usually include callousness, uncaring, and unemotional factors while two-factor (2F) models usually include callousness and uncaring factors, omitting the unemotional factor [15, 31, 32, 35]. The unemotional factor is typically omitted on the grounds that it is not useful for detecting psychopathic traits or externalizing problems [15, 31]. The second perspective pertains to whether or not the model employs a bifactor structure. A bifactor model encompasses a “general CU factor” in which all items are loaded, as well as other identified factors (such as callousness, unemotional and uncaring). Finally, some studies created a revised version of the ICU, usually eliminating some items to improve the model’s suitability to specific contexts. For example, while Moore et al. [35] created a revised ICU that omits only one item, Hawes et al. [31] short form excluded 12 items.

One possible explanation why different models have been suggested as the best-fit model is that the selection of items to improve the fit of the model is not based on the theoretical background. For example, when conducting confirmatory factor analysis (CFA), a combination of statistical and theoretical data should form the basis of the procedure of improvement of model fit using assumption errors among each item following modification indices. However, some studies have assumed a correlation of errors among models to suggest the best-fit model without sufficient explanation of its theoretical background [35]. In addition, individual studies investigate factor analysis using specifically chosen models without sampling all available models. For example, the Hawes’s 2F model [31] with a revised version of the ICU was the most-replicated model among the studies [21, 36]; however, some researchers suggested that other models were the best fit without examining Hawes’s 2F model [34, 35]. Therefore, there was no means of determining whether Hawes’s 2F model was the best fit for their data. The lack of sufficient investigation into the theoretical backgrounds and the tendency to not include all available models in the factor analyses in previous studies might have led to an unresolved situation whereby multiple best-fit models compete for primacy, and a standardized model remains elusive. To address this limitation, exhaustive analysis is needed using the full set of suggested factor models.

Another possible explanation for the inconsistent findings in the identification of the best-fit ICU model is the varying research settings and demographic characteristics of the samples, including distinct age ranges and gender distributions. For example, a previous study on the self-reported ICU among 13- to 18-year-old adolescents reported significant age-related differences in the ICU scores, and the author concluded that CU traits change over the course of development [15].

With regards to the research setting, some studies employed clinical settings or other environments populated by high-risk groups [21, 32]. Therefore, it is important to test whether those results can be generalized in a community sample to demonstrate the ICU’s utility in the general population.

Among studies that investigated factor structures of the parent-reported ICU, the total age range across all studies was from 6 to 20 years, though each focused on a specific developmental phase within this range, such as young children aged 6 to 12 years or 8 to 10 years, or adolescents aged 9 to 14 years old or from 14 to 20 years. No existing study has sampled both young children and adolescents and compared the results. It is well established that dynamic psychological development happens from childhood to adolescence. As such, it is a critical importance to enhance our understanding how age differences influence on the results of factor analyses of the parent-reported ICU.

## Study aims

In order to examine the superiority of any of the models proposed in previous studies, we performed an exhaustive investigation of such factorial models of the parent-reported ICU using a community sample with a wider age range from childhood to adolescence. In addition, we examined the psychometric property of our best-fit model by investigating the reliability, cross-sectional concurrent validity, and longitudinal predictive validity, with focus on the influences of age and gender. Moreover, reliability and validity comparisons between our best-fit model and the widely used 3FBF model [28,29] were conducted to examine the impact of certain transformations of the factor construct on the model’s reliability and validity.

## Materials and methods

### Participants

**Fig 1:**
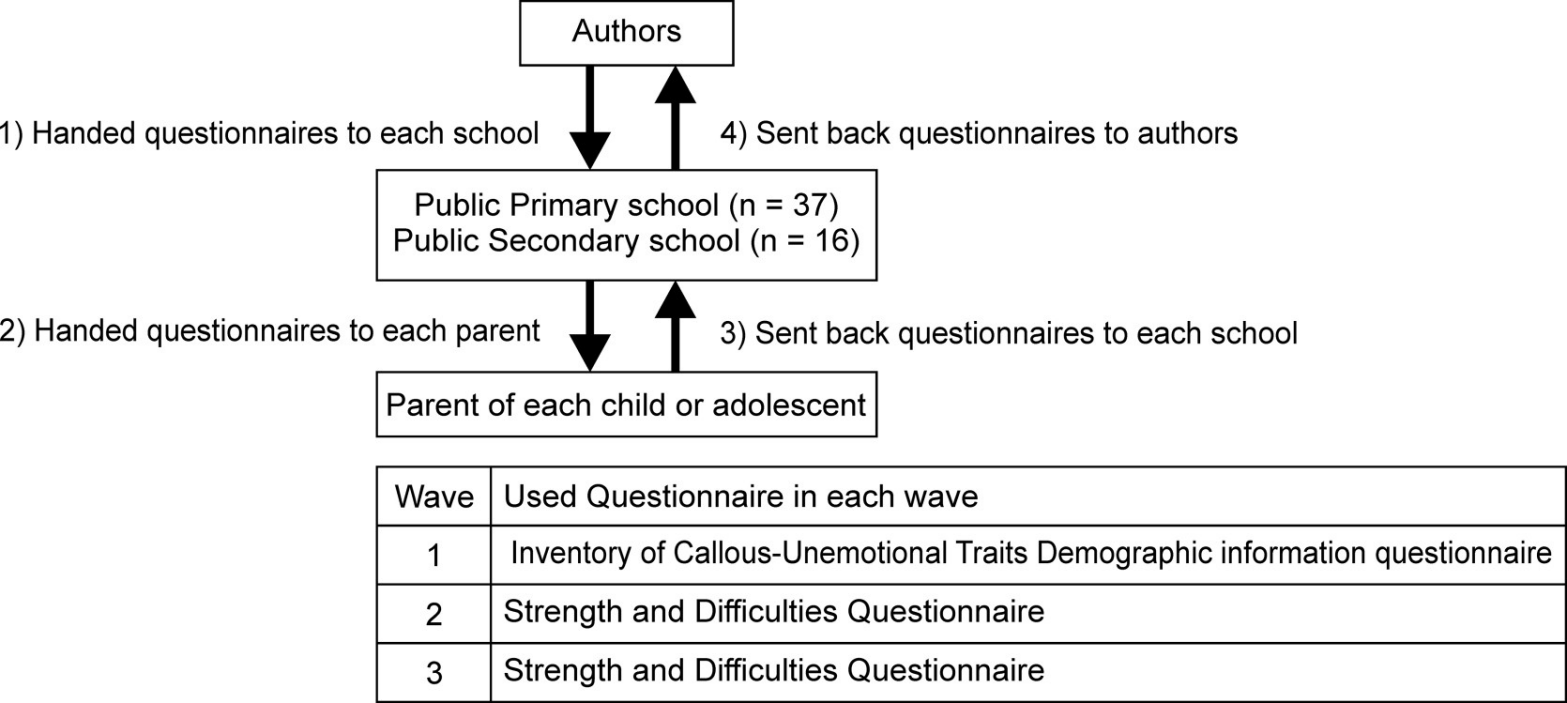
The procedure of data collecting.

The current study was conducted from 2015 to 2017 in Hirosaki City, Aomori Prefecture, Japan. Hirosaki City is located in the northern part of Honshu island in Japan and has an estimated population of 170,600, with 37 primary and 16 secondary schools. In terms of the economy, the average annual income is 2,764,330 Yen (24,698.72 USD), which is only marginally higher than the average income of Japan. We sent a set of questionnaires to all of the primary and secondary schools. Parent-reported questionnaires were distributed to the parents via their children. The first assessment period was in 2015 (Wave 1), and the second and third periods were in 2016 (Wave 2) and 2017 (Wave 3), respectively (Fig 1). During the first assessment period, we distributed questionnaires to parents whose children, aged 6 to 15 years (mean age = 11.00, SD = 2.58), were enrolled in compulsory education in Hirosaki city (n = 12,770) in the 1^st^ to 6^th^ grade of primary school or the 1^st^ to 3^rd^ grade of secondary school. In order not to interfere with participation and introduce selection bias (and consequently interference with our data), we did not make use of any incentive. In addition, we specifically instructed the teachers not to force or urge participants to complete the questionnaire.

A total of 10,936 (85.6%) parents answered the questionnaire, and after discarding incomplete questionnaires, data from a total of 9,797 completed questionnaires were used, including responses from 4,915 (50.2%) boys and 4,882 (49.8%) girls. We discarded the data as incomplete when even one missing answer was found on a questionnaire.

The responses from the parents of children in the 2^nd^ and 3^rd^ grade in secondary school during the first assessment (Wave 1) were excluded from the longitudinal analysis because they were outside of the age range of the study by the time Wave 2 and/or Wave 3 began. Based on our sampling method, roughly 90% of the children (aged 6 to 15 years) in the city were investigated during the study.

A total of 7,596 [3,801 males (50.04%) and 3,795 females (49.96%)] children from the 1^st^ to 6^th^ grade in primary school and the 1^st^ grade in secondary school were recruited for the longitudinal analysis. Ultimately, 6,100 (80.3%) parents of the children [2,999 males (49.2%) and 3,101 females (50.8%)] answered all questionnaires throughout all study periods, and the resulting data were included in this study. To ensure anonymity, we did not use any self-identifying data. We also explained to the parents that their completed questionnaires should be put into envelopes and self-sealed and that no other person should open them other than the researchers. Finally, we explained that the answers would be converted into numbers.

### Model identification

**Fig 2:**
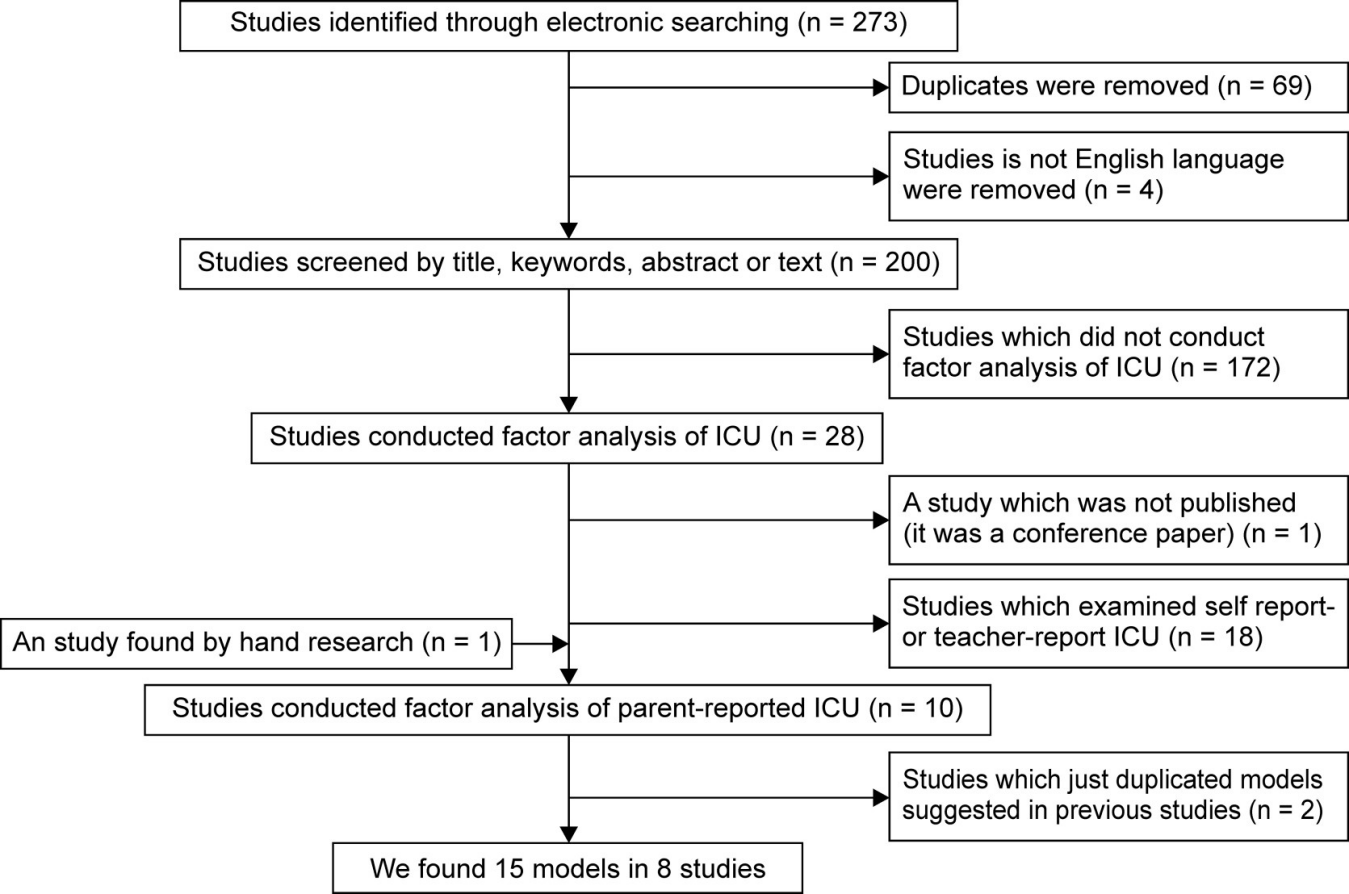
The procedure of model identification.

To find the factor models which were suggested in previous studies using a CFA for the parent-reported ICU, we adopted two strategies (Fig 2 and S1 Fig). First, a literature search of two databases, Web of Science and PubMed, was conducted. The period of the search was from January 1, 2010, to December 31, 2017; to the best of our knowledge, the first study of CFA for the parent-reported ICU was published in 2010 [27]. The search term “Inventory callous-unemotional traits” was used. Second, authors screened the studies by examining the titles, keywords, and abstracts. When it was unclear whether the article fit the inclusion criteria, the entire text was examined. All studies that examined the factor structure of the ICU by employing statistical methods such as CFA, exploratory factor analysis, and item response theory were included. Another inclusion criterion was that articles should be in English. In addition, the articles that were found in a manual search were also included.

### Measurement

#### Inventory of Callous-Unemotional traits (ICU)

The ICU comprises 24 items, each of which is rated on a four-point scale (0 = not true to 3 = very true), where a higher score indicates more CU traits [11]. In the original version, each score was a summation of the subscales: eleven items for callousness, eight items for uncaring, and five items for unemotional. The current study employed the parent-reported ICU [11]. The Japanese version of the ICU was developed using the translation and back-translation method, and its reliability was examined among Japanese community samples and reported as Cronbach’s’α = .74, Cohen’s d = .04 (unpublished data, available at https://kaken.nii.ac.jp/ja/file/KAKENHI-PROJECT-23659359/23659359seika.pdf).

#### Strengths and difficulties questionnaire (SDQ)

The SDQ is a short, well-validated screening questionnaire to evaluate a child’s psychological and behavioral problems [37]. The SDQ comprises five subscales (emotional symptoms, conduct problems, hyperactivity/inattention, peer relationship problems, and prosocial behavior). Each of the subscales has five items; thus, there are 25 items in total. In addition, the emotional symptoms and peer relationship problems subscale are classified as internalizing problems, whereas the conduct problems and hyperactivity/inattention subscales are classified as externalizing problems, alongside the prosocial behavior subscale [38]. Each of the items is scored on a 3-point scale (0 = not true, 1 = somewhat true, and 2 = certainly true). While a higher score on the other subscales indicates more severe behavioral problems, the prosocial behavior subscale is scored according to inverse criteria. The United Kingdom nationwide epidemiological study of psychopathology in children demonstrated good reliability and validity [39]. We employed the Japanese version of SDQ, which has been reported to have good reliability and validity [40].

### Statistical analyses

All statistical analyses in the study were conducted using Mplus 7 [41]. First, we conducted a CFA using a unidimensional model that includes all 24 items of the ICU. Second, a 3F model in which all items are loaded on three distinct factors was tested. Thirdly, we conducted CFAs with the models that were suggested in previous studies to determine which of them represented the best fit. Finally, by employing the selected best-fit model, we conducted multiple group analyses to investigate if the factor structure of the ICU was equivalent across gender (male and female) and age groups (a primary school-aged group of 6- to 12-year-olds and a secondary school-aged group of 13- to 15-year-olds). Models were estimated with mean and variance-adjusted weighted least squares estimation (WLSMV) for use with ordinal items [42]. Model fit was evaluated using the chi-square value, the comparative fit index (CFI) and the root mean square error of approximation (RMSEA). A general consensus on acceptable levels of fit indices is a values of .95 or more for CFI and a value of .08 or less for RMSEA [43]. Because we used WLSMV estimation, we conducted a corrected chi-square differences test using DIFFTEST [44]. The internal reliability of the questionnaires (that is, the ICU and SDQ) was assessed using Cronbach’s α, whereby α < .60 indicates insufficient fit, .60 to .69 indicates marginal fit, .70 to .79 indicates acceptable fit, .80 to .89 indicates good fit, and > .90 indicates excellent fit [45].

Finally, to investigate the cross-sectional and longitudinal concurrent validity of the ICU, we calculated the Pearson product-moment correlation coefficient between the ICU and SDQ. For this, we included the total difficulties score and each subscale from Wave 1 to evaluate the cross-sectional relationship between CU traits and internalizing problems (i.e., emotional symptoms), externalizing problems (i.e., conduct problems and hyperactivity/inattention), and prosocial behavior. We then computed a series of path models to examine the predictive ability of CU traits in Wave 1 with regard to internalizing and externalizing problems and prosocial behavior in Waves 2 and 3 (Table 1).

**Table 1:** The number of participants that transitioned to each Wave.

		Primary school						Secondary school		
		1st Grade	2nd Grade	3rd Grade	4th Grade	5th Grade	6th Grade	1st Grade	2nd Grade	3rd Grade
Wave 1	Male	519	518	524	518	506	607	609	575	506
	Female	513	494	569	549	553	562	555	575	545
	Total	1032	1012	1093	1067	1059	1169	1164	1150	1051
Wave 2	Male		466	467	483	466	447	511	545	480
	Female		467	453	510	489	502	483	498	493
	Total		933	920	993	955	949	994	1043	973
Wave 3	Male			431	426	431	420	388	429	474
	Female			437	423	472	455	430	426	458
	Total			868	849	903	875	818	855	932

### Ethics

The current study was approved by the Hirosaki University Graduate School of Medicine’s Committee of Medical Ethics. This study adhered to both the city’s and the committee’s information security policies concerning the protection of personal data. We mailed letters and information on the study to each child’s primary caregiver(s) to obtain informed consent, and we excluded data when primary caregivers indicated that they did not want their children to participate.

## Results

### Factor structure of the ICU

A detailed summary of the ways in which the articles were found and analyzed is presented in Fig 2, while Table 2 presents the model fit indices from the CFA in this study and a summary of previous studies including information about their sample and the factor structure of parent-reported ICU.

**Table 2:** Model fit statistics from the confirmatory factor analyses.

Factor structure	Model	Chi-Square Test of Model Fit	df^a^	RMSEA^b^ (90%CI)	CFI^c^	Sample	Item set	Study
**1 factor**	1	39208	252	0.12 (0.119 0.121)	0.661		All items loaded on a single CU factor	
**2 factor**	2	19427.21	251	0.085 (0.084 0.086)	0.833	1,078 community sample (50% male) of school-age (first-grade) children	Callous-Unemotional (10,12,4,9,11,18,6,20,2,21,22,7) Empathic-Prosocial (17,16,23,24,8,15,5,14,1,13,19,3)	Willoughby et al. [36]
	3	2725.44	53	0.059 (0.057 0.062)	0.944	250 boys exhibiting significant conduct problems 6–12 years	Callousness (11,6,12,4,21,18,9) Uncaring (8,16,5,24,17) eliminated of the item 1,2,3,7,10,13,14,19,20,22,23,15	Hawes et al. [31]
**2 factor bifactor**	4	13432.67	228	0.074 (0.073 0.075)	0.885	Same sample as Model 2	All items of Model 5 loaded on a general CU dimension as well as on three distinct factors	Willoughby et al. [36]
	5	22789.07	187	0.106 (0.105 0.108)	0.79	5092 16-year-oldtwin pairs	Callousness-Uncaring (3,4,5, 7, 8, 9, 11, 12, 13, 15, 16, 17, 18, 20, 21, 23, 24) Unemotional (1, 6, 14, 19, 22),and all items loaded on a general CU dimension as well as on two distinct factors Eliminated items 2, 10	Henry et al. [34]
	6	9186.1	228	0.104 (0.102 0.106)	0.788	Genetically informed community sample of 339 twin pairs (N = 678) between the ages of 9–14	Callous / Uncaring (2,3,4,5,7,8,9,10,11,12,13,15,16,17,18,20,21,23,24) Unemotional (1, 6,14,19,22), and all items loaded on a general CU dimension as well as on three distinct factors	Moore et al. [35]
**Restricted** **2 factor bifactor**	7	34749.86	241	0.116 (0.115 0.117)	0.7	Same sample as Model 6	Callous / Uncaring (3,7,11,15,20,23) Unemotional (1, 6,14,19,22), and all items load on a general CU dimension as well as on two distinct factors	Moore et al. [35]
**3 factor**	8	31900.44	249	0.109 (0.108 0.110)	0.725		ICU items loaded on three intercorrelated factors callousness (4,8,9,18,11,21,7,20,2,10,12) uncaring (15,23,16,3,17,24,13,5) unemotional (1,19,6,22,14)	
	9	27476.88	186	0.117 (0.116 0.118)	0.736	131 boys with ODD/CD (clinical) 6–12 years	Callousness/lack of guilt or remorse (21,9,4,17,18,16,12,8,24,13,5) Unconcerned about Performance (15,23,3,20,11) Unemotional (1,6,14,22,19) Eliminated items 2,7,10	Benesch et al. [32]
**3 bifactor**	10	21467.38	228	0.093 (0.092 0.094)	0.815	154 community adolescents between the ages of 14–20	All items loaded on a general CU dimension as well as on three distinct factors	Roose et al. [27]
	11	19172.09	187	0.097 (0.096 0.099)	0.824	Same sample as Model 5	Callousness-Uncaring (4, 7, 8, 9, 11, 12, 18, 20, 21) Uncaring (3, 5, 13, 15, 16, 17, 23, 24) Unemotional (1, 6, 14, 19, 22) , and all items loaded on a general CU dimension as well as on three distinct factors Eliminated items 2, 10	Henry et al. [34]
	12	6931.97	186	0.096 (0.095 0.097)	0.811	450 high risk 9-year-olds	Callousness (2,4,7,9,11,12,18,20,21) Uncaring (15,16,17,24) Unemotional (1,6,14,19,22), and all items loaded on a general CU dimension as well as on three distinct factors Eliminated items 10, 23	Waller et al. [21]
**Modified 3 factor**	13	12834.55	149	0.089 (0.088 0.091)	0.86	340 community sample aged 8–10 years	Callousness (4,7,9,11,12,18,20) Uncaring (3,5,13,15,16,17,23,24) Unemotional (1,14,19,22) Eliminated items 2,6,8,10,21	Gao & Zhang [33]
	14	10376.38	133	0.085 (0.084 0.086)	0.887	Same sample as Model 13	All items of Model 7 loaded on a general CU dimension as well as on three distinct factors	Gao & Zhang [33]
**Modified 3 factor with 8 pairs of correlated errors**	15	10817.47	141	0.084 (0.083 0.086)	0.882	Same sample as Model 13	To further improve the fit, modification indices for Model 5 were reviewed, and 8 pairs (20&15&23, 4&16, 23&15&3&5, 15&5&16&17) of error variables were correlated.	Gao & Zhang [33]
**2 factor bifactor**	16	1307.08	42	0.05 (0.047 0.052)	0.974	Same sample as Model 3	All items of Model 13 load on a general CU dimension as well as on two distinct factors	

^a^ df: Degrees of Freedom

^b^ RMSEA: Root Mean-Square Error of Approximation

^c^CFI: comparative fit index.

We labeled a unidimensional model with all items loading on a single ICU factor as Model 1 and a 3F model with all items loaded on three intercorrelated factors (callousness, uncaring, and unemotional) as Model 8. There was no study showing Model 1 or Model 8 as the best-fit, but we included them in our CFA because they are the basis models of the ICU. Among the 15 models, 1 model was a one-factor model, 2 models were 2F models [31, 36], 4 models were two-factor bifactor (2FBF) models [34, 35, 36], 2 models were 3F models [32], and 6 models were 3FBF models [21, 27, 33, 34].

The unidimensional model (Model 1), with all items loading on a single ICU factor, showed unsatisfactory fit, and the 3F model (Model 8) fit was significantly better (Δdf = 3, Δχ^2^ = 10969.469, *p* < .001); however, several fit indices were unacceptable. The 3FBF model (Model 10) showed a better fit than Model 8 (Δdf = 21, Δχ^2^ = 8095.045, *p* < .001), though it provided inadequate fit to the data.

Although direct comparisons were not possible for Models 2 through 15 due to different set of items, the 2F models (Models 2, 4 and 3, 6, 7) fit better than the 3F models (Models 8, 9, 10 and 13, 14, 12, 15); only one case, Models 5 and 11, showed an opposite result. Bifactor models (Models 10, 4, 11 and 6) fit better than the unifactor models (Models 8, 2, 13 and 7) when those models had the same factor structure (such as Models 8 and 10, Models 2 and 4, and Models 13 and 14). The best-fit model among these was Model 3 (a revised 2F model containing 12 items) and this was indicated by the CFI and RMSEA indices. Because using a bifactor model consistently showed a better fit than not, we employed the bifactor model with the Hawes’s model [31] (Model 16) and found that the model fit was better than Hawe’s model [31] (Δdf = 11, Δχ^2^ = 1109.66, *p* < .001; Model 16). Therefore, Model 16 was identified as the best-fit model in this study.

Using the best-fit model, which was a revised 2FBF model (Model 16), we investigated the influences of gender (male and female) and age group (primary school-aged group: 6- to 12-year-olds; secondary school-aged group: 13- to 15-year-olds) by conducting multiple group structural equation modeling comparing model fit when factor loadings and intercepts were fixed versus freed using the DIFFTEST procedure. We found that the fixed model showed a significantly better fit for both genders (Δdf = 21, Δχ^2^ = 37.43, *p* = .015) and age groups (Δdf = 21, Δχ^2^ = 101.18, *p* < .001), thus suggesting that factor loadings were similar across genders and age groups.

### Internal consistency of the best-fit model

We investigated the internal consistency and concurrent validity of Model 16 and the widely used Model 10 [28, 29]. Of these two, Model 16 showed the best fit in our CFA. The results of the internal consistency and cross-sectional bivariate correlations are summarized in Table 3. Acceptable internal consistency was found in total ICU scores, the callousness, and uncaring subscales in both the 2FBF and 3FBF models. However, only marginal internal consistency was found in the unemotional subscale in the 3FBF model.

**Table 3:** Internal reliability and cross-sectional bivariate correlations (total and subscale scores of the summed scores of the ICU).

	Model 16 (revised two-factor model of the ICU^a^)			Model 10 (Three-factor model of the ICU^a^)			
	Total	Callousness	Uncaring	Total	Callousness	Uncaring	Unemotional
Cronbach’s α	.752	.725	.756	.815	.704	.800	.614
**SDQ**^**b**^							
**Prosocial behavior**	-.494**	-.248**	-.521**	-.494**	-.202**	-.500**	-.327**
**Hyperactivity / inattention**	.425**	.323**	.342**	.478**	.373**	.447**	.108**
**Emotional symptoms**	.131**	.173**	.032**	.172**	.166**	.085**	.127**
**Conduct problem**	.471**	.356**	.421**	.451**	.358**	.407**	.119**
**Peer problems**	.274**	.234**	.197**	.314**	.207**	.226**	.253**
**Total difficulties score**	.448**	.376**	.327**	.499**	.389**	.413**	.209**

*p < .05

**p < .01

***p < .001.

^a^ICU: Inventory of callous-unemotional traits

^b^SDQ: Strength and difficulties questionnaire

The alpha values of the SDQ were as follows: prosocial behavior α = .729, hyperactivity/inattention α = .759, emotional symptoms α = .671, conduct problems α = .720, peer problems α = .620, and total difficulties score α = .796.

Moderate-to-strong associations were found between the total scores and the uncaring subscales of the ICU and the prosocial behavior subscale of the SDQ. On the other hand, the associations between the callousness subscales of the ICU and the prosocial behavior subscale of the SDQ were modest. Those results were consistent both in the 2FBF and 3FBF models.

Moderate associations were found between total scores, the callousness and uncaring subscales of the ICU in both the 2FBF and 3FBF models, and the hyperactivity/inattention and conduct problem subscales of the SDQ. In addition, even though the magnitude was less than in the case of callousness, uncaring, and total scores, there was a moderate association between the unemotional subscale of the 3FBF model and the hyperactivity/inattention and conduct problem subscales of the SDQ.

There were modest associations between the ICU total scores, callousness and uncaring subscales, and emotional symptoms subscale of the SDQ in both the 2FBF and 3FBF models.

### Construct validity of the 2FBF and 3FBF latent models

The results of the cross-sectional construct validity testing with latent models are presented in Table 4. In the cross-sectional analysis at Wave 1, higher general CU factor scores and higher callousness and uncaring scores were associated with a higher level of internalizing (emotional symptoms and peer problems subscales) and externalizing (hyperactivity/inattention and conduct problem subscales) problems and lower levels of prosocial behavior. These results were consistent in the 2FBF and 3FBF models. In particular, the general CU factor and conduct problem as well as prosocial behavior showed moderate to large associations. Within the 3FBF model, the unemotional scores were associated with higher emotional symptoms scores and *lower* level of hyperactivity/inattention and conduct problems scores, as well as *higher* levels of prosocial behavior scores.

**Table 4:** Regression models: General and specific ICU factor scores predicting subscales of the SDQ within 2FBF and 3FBF models.

	2FBF^a^ model of the ICU^b^				3FBF^c^ model of the ICU^b^				
	General CU factor	Callous specific factor	Uncaring specific factor	*R*^*2*^	General CU factor	Callous specific factor	Uncaring specific factor	Unemotional specific factor	*R*^*2*^
Wave 1 (2015)									
SDQ^d^									
Prosocial behavior	-.423***	-.153***	-.426***	.360***	-.582***	-.069***	-.056***	.047**	.345***
Hyperactivity / inattention	.370***	.211***	.228***	.233***	.443***	.304***	.327***	-.208***	.430***
Emotional symptoms	.133***	.179***	.159***	.080***	.067***	.205***	.100***	.296***	.144***
Conduct problem	.649***	.088***	.111**	.441***	.463***	.263***	.051**	-.254***	.351***
Peer problems	.239***	.163***	.108***	.096***	.213***	.196***	.102***	.237***	.150***
Total difficulties score	.436***	.306***	.043*	.284***	.438***	.330***	.197***	.073***	.345***
Wave 2 (2016)									
SDQ^d^									
Prosocial behavior	-.204***	-.070***	-.178***	.373***	-.167***	-.039***	-.057***	-.010	.350***
Hyperactivity / inattention	.061***	.057***	.039**	.495***	.110***	.099***	.070***	-.066***	.506***
Emotional symptoms	.032*	.043***	.035*	.347***	.020	.061***	.028*	.081***	.353***
Conduct problem	.252***	.040**	.051**	.414***	.138***	.092***	.021	-.096***	.402***
Peer problems	.076***	.044**	.036*	.347***	.070***	.060***	.057***	.049**	.333***
Total difficulties score	.079***	.077***	.043*	.504***	.082***	.077***	.038**	-.053***	.509***
Wave 3 (2017)									
SDQ^d^									
Prosocial behavior	-.120***	-.038**	.000	.425***	-.094***	-.037**	-.018	-.009	.403***
Hyperactivity / inattention	.019	.010	.017	.551***	.024	.020	.020	-.040*	.553***
Emotional symptoms	.037**	.019	-.014	.415***	.021	.022	.015	.030	.415***
Conduct problem	.160***	.002	.047**	.474***	.071***	.022	-.009	-.054**	.466***
Peer problems	.050***	.022	.012	.410***	.048***	.021	-.012	-.013	.410***
Total difficulties score	.029*	.013	.005	.565***	.019	.020	-.004	-.020	.570***

*p < .05

**p < .01

***p < .001.

^a^ 2FBF: two-factor bifactor

^b^ ICU: Inventory of callous-unemotional traits

^c^ 3FBF:three-factor bifactor

^d^ SDQ: Strength and difficulties questionnaire

The results of the longitudinal regression analysis are shown in Table 4. In the longitudinal analysis, we adopted the autoregressive models that controlled the past levels on the outcome (i.e. stability effects). For example, the SDQ prosocial behavior subscale scores at Wave 2 were controlled by the SDQ prosocial behavior subscale scores at Wave 1, and the SDQ prosocial behavior subscale scores at Wave 3 were controlled by the SDQ prosocial behavior subscale scores at Waves 1 and 2.

The general CU factor scores significantly predicted the increase of conduct problems, emotional symptoms and peer problems scores, and a decrease of prosocial behavior scores in the SDQ through Waves 2 to 3. Higher hyperactivity/inattention scores on the SDQ were also predicted by the general CU factor scores but it found only at Wave 2 but not at Wave 3. In the 3FBF model, the general CU factor scores predicted conduct and peer problem scores at Waves 2 and 3, whereas it predicted hyperactivity/inattention scores only at Wave 2; emotional symptoms scores were not predicted at Wave 2 nor Wave 3. In particular, the general CU factor scores of the 2FBF model strongly predicted the declines in prosocial behavior scores and the increases in conduct problem scores compared to other SDQ subscales. A similar tendency was shown in the general CU factor of the 3FBF model, but the influence of prosocial behavior scores and conduct problem scores in Wave 3 were lower compared to the 2FBF model.

The callousness specific factor scores in both the 2FBF and 3FBF models predicted significantly higher conduct problems, hyperactivity/inattention, peer problems, emotional symptoms scores, and lower prosocial behavior scores on the SDQ at Wave 2. However, only lower prosocial behavior score was predicted at Wave 3 in both models.

The uncaring specific factor scores in the 2FBF model predicted significantly higher conduct problems, hyperactivity/inattention, emotional symptoms, and peer problems scores, as well as a lower prosocial behavior score at Wave 2; however, it predicted only the conduct problems score at Wave 3. In the 3FBF model, the uncaring specific factor scores predicted all factors except for conduct problems score and did not predict any subscale scores of the SDQ at Wave 3.

The unemotional specific factor scores in 3FBF model *negatively* predicted hyperactivity/inattention and conduct problems scores on the SDQ and positively predicted higher peer problems and emotional problems scores on the SDQ at Wave 2 but not at Wave 3. However, there was no relationship between the unemotional factor score and prosocial behavior score on the SDQ in Wave 2 nor Wave 3.

We conducted a regression analysis to examine the relationship between the ICU summed total scores and each subscale of the SDQ because the latent models, in which a factor does not mean a summed total score, cannot be adopted for practical use of the ICU, and the summed SDQ total difficulties scores can be used in this case (see S1 Table). The pattern of the findings broadly mirrored the results of latent model frameworks in both the 2FBF and the 3FBF.

## Discussion

In this study, we investigated the best-fit model for the parent-reported ICU by conducting CFA among 15 models that were suggested in previous studies, and we then used the best-fit model from the CFA to investigate the concurrent and predictive validity of the parent-reported ICU with a community sample of 6- to 15-year-olds.

### Confirmatory factor analysis

The current study found that Hawes’s 2F model using the revised versions of ICU [31], which included only 12 items that were primarily from the callousness and uncaring factors, showed the best fit among the models suggested in previous studies. This result is consistent with previous studies using samples of primary school- and pre-school-aged children [21, 28].

It is noteworthy that a bifactor solution to Hawes’ 2F model showed the better fit. Hawes’ 2F model was originally shown as the best-fit model based on studies examining children aged 6 to 12 years who were exhibiting significant conduct problems [31], and Waller et al. [21] validated this among 9-year-old children with high-risk factors. Later, Kimonis et al. [23] confirmed the model’s validity among preschool children aged 3 to 6 years in a community setting. The current study is the first to have examined Hawes’s revised ICU in a primary and secondary school-aged community sample, and the results demonstrated that the 2F model showed the best fit within this group. Moreover, the findings showed the suitability of fit of the 2FBF model and did not demonstrate a significant difference across age groups and genders.

Our study was conducted with a large community sample and a broad age range of 6 to 15 years. Examining a broad age band has been widely suggested as inappropriate for capturing patterns across the sample due to developmental differences between groups of children even relatively close in age. For example, a previous study reported inconsistent findings in a CFA of the ICU across age groups (13–14 years old, 15–16 years old, and 17–18 years old) and attributed the variations in the findings to changes in a normative level of CU traits over the course of development [15]. Our results indicated that there was no influence of age on the factor structure of the ICU. This echoes Gao and Zhang’s study [33], which found no differences in the factor structure or levels of the factors in the parent-reported ICU in 8 to 10-year old boys and girls. Also, Pihet et al [46] reported the overall utility of the ICU for assessing CU traits without regard to age, gender, or institutionalized status.

One possible explanation for the discrepancy between our study and previous findings might be differences between informers. For instance, Essau et al. [15] examined data from the self-reported ICU, whereas the current study employed the parent-reported ICU. The ability to evaluate oneself correctly relies on cognitive development; therefore, the results of the self-reported questionnaire might be less consistent than those reported by adults [47]. A previous study investigated the effects of age on the results of the self-reported SDQ and found that older adolescents reported more emotional symptoms and prosocial behavior than younger children [48], thus suggesting that a child’s self-assessment of CU traits might be impacted by age differences. However, even though a child’s CU traits change over time, parents using the parent-reported ICU can objectively capture these shifts in a manner that aligns with normative levels (i.e., the behavior of other children of the same age), which also changes over time. In fact, a previous review demonstrated that the stability of the CU traits was higher when observed by parents compared to self-report [6].

It is important to note that while the short form of the ICU has been previously found to resolve various problems reported previously for the ICU [47], it does not include items that have been selected to assess for DSM-5 specifier criterion “concerned about performance at school, work, or in other important activities” as captured in the work of Kimonis et al. [49]. Also, the presence of a single item [30] or none [47] that assesses unemotionality also implies that the short-form ICU does not allow a thorough assessment of the DSM-5 specifier criterion ‘Shallow and Deficient Affect’. Therefore, while the ICU content may provide a continuous measure of CU traits [50], the items selected for the short-form ICU limit how CU traits may be assessed as defined by the DSM-5 specifier. These observations underscore the need to be cautious in generalizing the results of our best-fit model in relation to children who meet the criteria for the DSM-5 LPE specifier.

### Construct validity

First, we should be cautious in interpreting the results of regression models with small effect sizes, as the statistically significant results observed in this study could have been due to its large sample size. Aside from statistical significance, Ferguson [51] showed the minimum level of “practical” significance for effect sizes with 0.20 for the standard regression coefficient. However, in longitudinal analysis based on the autoregressive model, it is necessary to lower standards of meaningful effect size compared to that of the cross-sectional analysis. This is because controlling past levels on the outcome (stability effects) often removes a large portion of variance in the outcome that was shared with predictors. For meaningful effect sizes in the longitudinal autoregressive model, β > .05 was recommended as one criterion [52]. According to these standards, in both the 2FBF model and 3FBF model, general CU factor scores can be interpreted as showing a meaningful relationship with conduct problems, hyperactivity/inattention, peer problems, and prosocial behavior scores at Wave 1. Furthermore, general CU factor scores have been shown to be able to predict the decrease in prosocial behavior and an increase in conduct problem scores at a meaningful level even in Waves 2 and 3. These results demonstrate the ability of the general CU factor to not only predict concurrent conduct problems and lower social behaviors but also to project their development two years later despite the controlled influence of the stability effects. The results are also consistent with a previous study demonstrating the longitudinal predictive validity of parent-reported ICU [29].

In the 2FBF model, a higher callous specific factor score had a meaningful association with a higher hyperactivity/inattention score at Waves 1 and 2. A higher uncaring specific factor score showed a meaningful association with a higher prosocial behavior score at Waves 1 and 2. However, no specific factor was able to predict the subscale of SDQ in Wave 3 at a meaningful level. In the 3FBF model, the callous/uncaring specific factor scores showed meaningful associations with a broader area of subscales in SDQ compared to the 2FBF model at Waves 1 and 2. However, not predicting any SDQ subscale of Wave 3 was similar to the 2FBF model. Thus, the results of this study indicate that the general factor is more predictive than the specific factor in predicting long-term externalizing problems. The higher predictive power of the general factor compared to the specific factor has also been suggested in studies of high-risk 9-year-old children [21]. On this note, the results of this study suggest that similar results can be obtained for a wider age range of community samples.

The unemotional factor in the 3FBF model was negatively related to conduct problems and hyperactivity/inattention subscale scores through all assessment periods from Waves 1 to 3. This result aligns with previous findings that higher unemotional scores are related to lower aggression and rule-breaking scores [21]. If we consider that one of the expected roles of the ICU is to detect severe CD, the characteristics of the unemotional factor, which relates negatively to externalizing problems, might not be ideal for its purposes. Thus, items included in the unemotional factor did not appear to operate as intended in the nomological basis of CU behavior and thus may not be clinically or conceptually useful. These points suggest that in using the ICU for assessment, the most meaningful and reliable predictive validity may be derived via the use of a latent general or summed total score.

From the results described above, we conclude that first, the parent-rated ICU may be used to assess children’s CU traits, and this is predictive of future issues such as conduct problems and less prosocial behavior. Hence, this provides an avenue for early detection and diagnosis of CU traits, with the implication that early interventions can be sought (from clinical professionals) and implemented. It is important to note that psychopathy is an early-appearing risk factor for severe and chronic violence, which accounts for a large part of the societal burden to the public health and criminal justice systems. Hence, the use of the parent-reported ICU can facilitate not only the early detection of CU traits, but it can help prevent some of the potential consequences of violence-associated behavior [53]. Second, the revised version of ICU (with 12 items) works as well as the original ICU, which suggests that the former is less costly in terms of time and material resources. In addition, considering that the unemotional factor showed a negative relationship with future conduct problems and hyperactivity/inattention, employing the revised version by omitting all but one item of the unemotional factors is more efficient in predicting higher CU traits, which are regarded as a marker of severe CD. Furthermore, our results suggested that in using the ICU for assessment, the most meaningful and reliable predictive validity is derived via the use of a latent general CU score.

### Study strengths and limitations

The current study has three main strengths. First, this is the first study to conduct an exhaustive factor analysis of the parent-reported ICU. Second, this is the first trial to conduct a two-year longitudinal study examining the predictive validity of the ICU. Third, we employed a large community sample with a wider age range (6–15 years) than previous studies.

There were also several limitations in the current study. First, we had some attrition in our sample over the two years of the study due to causes ranging from a child’s absence from school on days designated for our study, to children’s refusal to continue attending the sessions. There was no information provided regarding why some of the children dropped out; therefore, it is possible that the results were influenced by differences in the characteristics of children who attended all study periods and those who did not. For example, the correlation between significant negative academic behavior such as decreased school attendance and CU traits has been previously reported [54]. Thus, it is possible that some of the children (if not all) who completed the study might have less CU traits than those that did not. This suggests the possibility that our study might have investigated a significant proportion of “healthier” sample.

Second, though the average income in Hirosaki is almost the same as that of the whole of Japan, the social, educational, and economic dynamics of Hirosaki are not representative of the whole of Japan because Hirosaki is a medium-sized city in a rural area. Therefore, care should be taken in generalizing our results to the entire Japanese population.

Finally, only the parent-reported ICU was employed in the current study. While using the self-reported ICU might pose some challenges to younger children; further studies including self- and teacher- reported data should be conducted to compare the influences of different informers in order to increase the accuracy of the ICU’s CFA.

## Conclusions

The current study is the first to 1) conduct a factor analysis of the parent-reported ICU with the CFA investigating all models suggested in previous studies and 2) compare the concurrent and predictive validity of the original widely used model and the best-fit model determined in our study. We demonstrated that the best-fit model of the ICU was the 2FBF model using the revised version (with 12 items) developed by Hawes et al. [31] among a school-aged community sample. In addition, we presented the concurrent and predictive validity of the revised ICU based on significant relationships with other variables in a cross-sectional and longitudinal analysis.

## Supporting information

S1 Table Regression models: Summed ICU total and subscale scores predicting subscales of the SDQ within 2 factor and 3 factor models *p < .05; **p < .01; ***p < .001. (DOCX)

S1 Fig PRISMA flow diagram. (TIF)

## References

[1] Frick PJ. Developmental pathways to conduct disorder. Child Adolesc Psychiatry Clin N Am. 2006;15(2):311–331. 10.1016/j.chc.2005.11.003 16527658

[2] Scott S, Knapp M, Henderson J, Maughan B. Financial cost of social exclusion: follow up study of antisocial children into adulthood. BMJ. 2001;323:191 10.1136/bmj.323.7306.191 11473907 PMC35269

[3] Odgers CL, Caspi A, Broadbent JM, Dickson N, Hancox RJ, Harrington H, et al Prediction of differential adult health burden by conduct problem subtypes in males. Arch Gen Psychiatry. 2007;64(4):476–484. 10.1001/archpsyc.64.4.476 17404124

[4] Frick PJ, Ellis M. Callous-unemotional traits and subtypes of conduct disorder. Clin Child Fam Psychol Rev. 1999;2(3):149–168. 11227072 10.1023/a:1021803005547

[5] Frick PJ, Cornell AH, Barry CT, Bodin SD, Dane HE. Callous-unemotional traits and conduct problems in the prediction of conduct problem severity, aggression, and self-report of delinquency. J Abnorm Child Psychol. 2003;31(4):457–470. 12831233 10.1023/a:1023899703866

[6] Frick PJ, White SF. Research review: the importance of callous‐unemotional traits for developmental models of aggressive and antisocial behavior. J Child Psychol Psychiatry. 2008;49(4):359–375. 10.1111/j.1469-7610.2007.01862.x 18221345

[7] Frick PJ, Ray JV, Thornton LC, Kahn RE. Can callous-unemotional traits enhance the understanding, diagnosis, and treatment of serious conduct problems in children and adolescents? A comprehensive review. Psychol Bull. 2014;140(1):1–57. 10.1037/a0033076 23796269

[8] Diagnostic and statistical manual of mental disorders (5th Ed). Washington DC: American Psychiatric Association; 2013.

[9] McDonough-Caplan HM, Beauchaine TP. Conduct disorder: a neurodevelopmental perspective In: Martel MM, editor. Developmental pathways to disruptive, impulse-control and conduct disorders. Academic Press; 2018 p. 53–89,

[10] Jambroes T, Jansen LM, Vermeiren RR, Doreleijers TA, Colins OF, Popma A. The clinical usefulness of the new LPE specifier for subtyping adolescents with conduct disorder in the DSM 5. Eur Child Adolesc Psychiatry. 2016;25(8):891–902. 10.1007/s00787-015-0812-3 26725044

[11] Frick PJ. The Inventory of Callous-Unemotional traits: Unpublished rating scale. University of New Orleans 2004 Available from: http://labs.uno.edu/developmental-psychopathology/ICU.html.

[12] Frick PJ, Hare RD. Antisocial process screening device: APSD, Multi-Health Systems Toronto 2001 Available from: http://labs.uno.edu/developmental-psychopathology/APSD.html.

[13] Frick PJ, Bodin SD, Barry CT. Psychopathic traits and conduct problems in community and clinic-referred samples of children: further development of the psychopathy screening device. Psychol Assess. 2000;12(4):382–393. 11147105

[14] Frick PJ, O'brien BS, Wootton JM, McBurnett K. Psychopathy and conduct problems in children. J Abnorm Psychol. 1994;103(4):700–707. 7822571 10.1037//0021-843x.103.4.700

[15] Essau CA, Sasagawa S, Frick PJ. Callous-unemotional traits in a community sample of adolescents. Assessment. 2006;13(4):454–469. 10.1177/1073191106287354 17050915

[16] Ciucci E, Baroncelli A, Franchi M, Golmaryami FN, Frick PJ. The association between callous-unemotional traits and behavioral and academic adjustment in children: further validation of the inventory of callous-unemotional traits. J Psychopathol Behav Assess. 2014;36(2):189–200.

[17] Feilhauer J, Cima M, Arntz A. (2012). Assessing Callous Unemotional traits across different groups of youths: further cross-cultural validation of the Inventory of Callous-Unemotional traits. Int J Law Psychiatry. 2012;35(4):251–262. 10.1016/j.ijlp.2012.04.002 22575180

[18] Fanti KA, Frick PJ, Georgiou S. Linking callous unemotional traits to instrumental and non-instrumental forms of aggression. J Psychopathol Behav Assess. 2009;31(4): 285–298.

[19] Berg JM, Lilienfeld SO, Reddy SD, Latzman RD, Roose A, Craighead LW, et al The inventory of callous and unemotional traits: a construct- validational analysis in an at-risk sample. Assessment. 2013;20 5: 532–544. 10.1177/1073191112474338 23344913

[20] Bhanwer AK, Viljoen JL, Shaffer CS, Douglas KS. The Inventory of Callous-Unemotional Traits: Reliability, Convergent Validity, and Predictive Validity for Reoffending in Adolescents on Probation. Int J Forensic Ment Health. 2019;18 (2)111–123.

[21] Waller R, Wright AG, Shaw DS, Gardner F, Dishion TJ, Wilson MN, et al Factor structure and construct validity of the parent-reported Inventory of Callous-Unemotional Traits among high-risk 9-year-olds. Assessment. 2015;22(5):561–580. 10.1177/1073191114556101 25371449 PMC4937624

[22] Viding E, Simmonds E, Petrides KV, Frederickson N. The contribution of callous‐unemotional traits and conduct problems to bullying in early adolescence. J Child Psychol Psychiatry. 2009;50 4: 471–481. 10.1111/j.1469-7610.2008.02012.x 19207635

[23] Kimonis ER, Fanti KA, Anastassiou-Hadjicharalambous X, Mertan B, Goulter N, Katsimicha E. Can callous-unemotional traits be reliably measured in preschoolers? J Abnorm Child Psychol. 2016;44(4):625–638. 10.1007/s10802-015-0075-y 26344015

[24] Ezpeleta L, Osa ND, Granero R, Penelo E, Domènech JM. Inventory of callous-unemotional traits in a community sample of preschoolers. J Clin Child Adolesc Psychol. 2013;42 1: 91–105. 10.1080/15374416.2012.734221 23095075

[25] Somech LY, Elizur Y. Promoting self-regulation and cooperation in pre-kindergarten children with conduct problems: a randomized controlled trial. J Am Acad Child Adolesc Psychiatry. 2012;51(4):412–422. 10.1016/j.jaac.2012.01.019 22449647

[26] Tye C, Bedford R, Asherson P, Ashwood KL, Azadi B, Bolton P, et al Callous-unemotional traits moderate executive function in children with ASD and ADHD: a pilot event-related potential study. Dev Cogn Neurosci. 2017;26:84–90. 10.1016/j.dcn.2017.06.002 28654838 PMC5569583

[27] Roose A, Bijttebier P, Decoene S, Claes L, Frick PJ. Assessing the affective features of psychopathy in adolescence: a further validation of the inventory of callous and unemotional traits. Assessment. 2010;17(1):44–57. 10.1177/1073191109344153 19797326

[28] Kimonis ER, Cross B, Howard A, Donoghue K. Maternal care, maltreatment and callous-unemotional traits among urban male juvenile offenders. J Youth Adolesc. 2013;42(2):165–177. 10.1007/s10964-012-9820-5 23054349

[29] Herrington LL, Barry CT, Loflin DC. Callous-unemotional traits, narcissism, and behavioral history as predictors of discipline problems in an adolescent residential program. Resid Treat Child Youth. 2014;31(4):253–265. 10.1080/0886571X.2014.958341

[30] Horan JM, Brown JL, Jones SM, Aber JL. Assessing invariance across sex and race/ethnicity in measures of youth psychopathic characteristics. Psychol Assess. 2015;27(2):657–668. 10.1037/pas0000043 25383582 PMC5497990

[31] Hawes SW, Byrd AL, Henderson CE, Gazda RL, Burke JD, Loeber R, et al Refining the parent-reported inventory of callous-unemotional traits in boys with conduct problems. Psychol Assess. 2014;26(1):256–266. 10.1037/a0034718 24188153

[32] Benesch C, Görtz-Dorten A, Breuer D, Döpfner M. Assessment of callous-unemotional traits in 6 to 12 year-old children with Oppositional Defiant Disorder/Conduct Disorder by parent ratings. J Psychopathol Behav Assess. 2014;36(4):519–529.

[33] Gao Y, Zhang W. Confirmatory factor analyses of self- and parent-report Inventory of Callous-Unemotional Traits in 8- to 10-year-olds. J Psychopathol Behav Assess. 2016;38(3):331–340. 10.1007/s10862-015-9527-5 28255197 PMC5328416

[34] Henry J, Pingault JB, Boivin M, Rijsdijk F, Vidinh E. Genetic and environmental aetiology of the dimensions of Callous-Unemotional traits. Psychol Med. 2016;46(2):405–414. 10.1017/S0033291715001919 26456336 PMC4682480

[35] Moore AA, Carney D, Moroney E, Machlin L, Towbin KE, Brotman MA, et al The Inventory of Callous-Unemotional Traits (ICU) in children: reliability and heritability. Behav Genet. 2017;47(2):141–151. 10.1007/s10519-016-9831-1 27909830 PMC5305430

[36] Willoughby MT, Mills-Koonce WR, Waschbusch DA, Gottfredson NC, Family Life Project Investigators. An Examination of the Parent Report Version of the Inventory of Callous-Unemotional Traits in a Community Sample of First-Grade Children. Assessment. 2015;22(1):76–85. 10.1177/1073191114534886 24820529 PMC8011843

[37] Goodman R. The Strengths and Difficulties Questionnaire: a research note. J Child Psychol Psychiatry. 1997;38(5):581–586. 9255702 10.1111/j.1469-7610.1997.tb01545.x

[38] Goodman A, Lamping DL, Ploubidis GB. When to use broader internalising and externalising subscales instead of the hypothesised five subscales on the Strengths and Difficulties Questionnaire (SDQ): data from British parents, teachers and children. J Abnorm Child Psychol. 2010;38(8):1179–1191. 10.1007/s10802-010-9434-x 20623175

[39] Goodman R. Psychometric properties of the strengths and difficulties questionnaire. J Am Acad Child Adolesc Psychiatry. 2001;40(11):1337–1345. 10.1097/00004583-200111000-00015 11699809

[40] Matsuishi T, Nagano M, Araki Y, Tanaka Y, Iwasaki M, Yamashita Y, et al Scale properties of the Japanese version of the Strengths and Difficulties Questionnaire (SDQ): a study of infant and school children in community samples. Brain Dev. 2008;30(6):410–415. 10.1016/j.braindev.2007.12.003 18226867

[41] Muthén LK, Muthén BO. Mplus user’s guide (7th ed). Los Angeles: Muthén & Muthén; 1998–2010.

[42] Flora D, Curran P. An empirical evaluation of alternative methods of estimation for confirmatory factor analysis with ordinal data. Psychol Methods. 2004;9(4):466–491. 10.1037/1082-989X.9.4.466 15598100 PMC3153362

[43] Hu LT, Bentler PM. Cutoff criteria for fit indexes in covariance structure analysis: Conventional criteria versus new alternatives. Struct Equ Modeling. 1999;6(1):1–55. 10.1080/10705519909540118

[44] Muthén LK, Muthén BO. Mplus. Los Angeles, CA: Muthén & Muthén; 2014.

[45] Barker C, Pistrang N, Elliot R. Research methods in clinical and counselling psychology. New Jersey: John Wiley & Sons; 1994.

[46] Pihet S, Etter S, Schmid M, Kimonis ER. Assessing callous-unemotional traits in adolescents: Validity of the inventory of callous-unemotional traits across gender, age, and community/institutionalized status. J Psychopathol Behav Assess. 2015;37(3):407–421.

[47] Colins OF, Andershed H, Hawes SW, Bijttebier P, Pardini DA. Psychometric properties of the original and short form of the Inventory of Callous-Unemotional Traits in detained female adolescents. Child Psychiatry Hum Develop. 2016 47(5):679–90. 10.1007/s10578-015-0601-8 PMC4996871 26493393

[48] Becker A, Hagenberg N, Roessner V, Woerner W, Rothenberger A. Evaluation of the self-reported SDQ in a clinical setting: do self-reports tell us more than ratings by adult informants? Eur Child Adolesc Psychiatry. 2004;13 Suppl 2: II17–24. 15243782 10.1007/s00787-004-2004-4

[49] Kimonis ER, Fanti KA, Frick PJ, Moffitt TE, Essau C, Bijttebier P, et al Using self‐reported callous‐unemotional traits to cross‐nationally assess the DSM‐5 ‘With Limited Prosocial Emotions’ specifier. J Child Psychol Psych. 2015;56(11);1249–1261. 10.1111/jcpp.12357 25360875

[50] Ray JV, Frick PJ, Thornton LC, Steinberg L, Cauffman E. Positive and negative item wording and its influence on the assessment of Callous-Unemotional Traits. Psychol Assess. 2016 28(4) 394–404. 10.1037/pas0000183 26121386

[51] Ferguson CJ. An effect size primer: a guide for clinicians and researchers. Professional Psychology: Research and Practice. 2009;40(5):532.

[52] Adachi P, Willoughby T. Interpreting effect sizes when controlling for stability effects in longitudinal autoregressive models: Implications for psychological science. European Journal of Developmental Psychology. 2015;12(1):116–128.

[53] Reidy DE, Kearns MC, DeGue S, Lilienfeld SO, Massetti G, Kiehl KA. Why psychopathy matters: Implications for public health and violence prevention. Aggress Violent Behav. 2015;24:214–25. 10.1016/j.avb.2015.05.018 29593448 PMC5868481

[54] Sourander A, Jensen P, Ronning JA, Niemela S, Helenius H, Sillanmaki L, et al What is the early adulthood outcome for boys who bully or are bullied in childhood: the Finnish “From a Boy to a Man” Study. Pediatrics. 2007;120:397–404. 10.1542/peds.2006-2704 17671067

