# Supplementary material for: The factor structure and construct validity of the parent-reported Inventory of Callous-Unemotional Traits among school-aged children and adolescents
Source: PLoS One. 2019 Aug 16;14(8):e0221046. doi: 10.1371/journal.pone.0221046 (PMC6697337; doi:10.1371/journal.pone.0221046)
Supplement: S1 Table — (DOCX) [file pone.0221046.s001.docx]

**S1 Table. Regression models: Summed ICU total and subscale scores predicting subscales of SDQ with in 2 factor and 3 factor models**

|  | 2 factor | | | | | 3 factor | | | | | |
| --- | --- | --- | --- | --- | --- | --- | --- | --- | --- | --- | --- |
|  | Regression Mode l | | Regression Mode 2 | | | Regression Mode 3 | | Regression Mode 4 | | | |
|  | ICU Total score | *R^2^* | Callous  specific factor | Uncaring  specific factor | *R^2^* | ICU Total score | *R^2^* | Callous  specific factor | Uncaring  specific factor | Unemotional  specific factor | *R^2^* |
| **wave 1 (2015)** |  |  |  |  |  |  |  |  |  |  |  |
| SDQ |  |  |  |  |  |  |  |  |  |  |  |
| Prosocial behavior | -.470*** | .221*** | -.148*** | -.448*** | .250*** | -.478*** | .229*** | -.143*** | -.403*** | -.075** | .252*** |
| Hyperactivity / inattention | .409*** | .167*** | .291*** | .238*** | .169*** | .449*** | .202*** | .352*** | .343*** | -.126*** | .262*** |
| Emotional symptoms | .134*** | .018*** | .186*** | -.007 | .034*** | .156*** | .024*** | .144*** | -.014 | .091*** | .034*** |
| Conduct problem | .416*** | .173*** | .294*** | .244** | .175*** | .418*** | .175*** | .328*** | .307** | -.103*** | .221*** |
| Peer problems | .249*** | .062*** | .210*** | .115*** | .067*** | .276*** | .076*** | .162*** | .075*** | .144*** | .082*** |
| Total difficulties score | .428*** | .183*** | .346*** | .210*** | .194*** | .462*** | .213*** | .351*** | .258*** | .004 | .238*** |
| **wave 2 (2016)** |  |  |  |  |  |  |  |  |  |  |  |
| SDQ |  |  |  |  |  |  |  |  |  |  |  |
| Prosocial behavior | -.134*** | .345*** | -.057*** | -.118*** | .343*** | -.135*** | .341*** | -.051*** | -.109*** | -.025*** | .343*** |
| Hyperactivity / inattention | .072*** | .484*** | .056*** | .036** | .499*** | .079*** | .499*** | .079*** | .064*** | -.032** | .502*** |
| Emotional symptoms | .028** | .342*** | .043*** | -.003 | .347*** | .036*** | .347*** | .040*** | .002 | .010 | .347*** |
| Conduct problem | .089*** | .380*** | .067*** | .051*** | .388*** | .091*** | .388*** | .084*** | .073*** | -.030** | .391*** |
| Peer problems | .075*** | .324*** | .056*** | .041*** | .326*** | .080*** | .327*** | .047*** | .047*** | .013 | .327*** |
| Total difficulties score | .059*** | .498*** | .049*** | .027** | .505*** | .064*** | .506*** | .062*** | .044*** | -.016 | .507*** |
| **wave 3 (2017)** |  |  |  |  |  |  |  |  |  |  |  |
| SDQ |  |  |  |  |  |  |  |  |  |  |  |
| Prosocial behavior | -.083*** | .405*** | -.044*** | -.063*** | .398*** | -.079*** | .397*** | -.041*** | -.047*** | -.017 | .397*** |
| Hyperactivity / inattention | .004 | .542*** | .015 | -.009 | .553*** | .007 | .553*** | .023* | .012 | -.023* | .553*** |
| Emotional symptoms | .026** | .415*** | .019 | .014 | .416*** | .025** | .416*** | .020 | .015 | -.005 | .416*** |
| Conduct problem | .044*** | .466*** | .026** | .031** | .462*** | .040*** | .462*** | .031** | .045*** | -.021* | .463*** |
| Peer problems | .044*** | .412*** | .026** | .030** | .410*** | .040*** | .410*** | .037*** | .020 | -.005 | .410*** |
| Total difficulties score | .018* | .567*** | .010 | .011 | .568*** | .014 | .568*** | .020* | .021* | -.024** | .569*** |

*p<.05;
**p<.01;
***p<.001.
