# Supplementary figures and images for: The factor structure and construct validity of the parent-reported Inventory of Callous-Unemotional Traits among school-aged children and adolescents
Source: PLoS One. 2019 Aug 16;14(8):e0221046. doi: 10.1371/journal.pone.0221046 (PMC6697337; doi:10.1371/journal.pone.0221046)

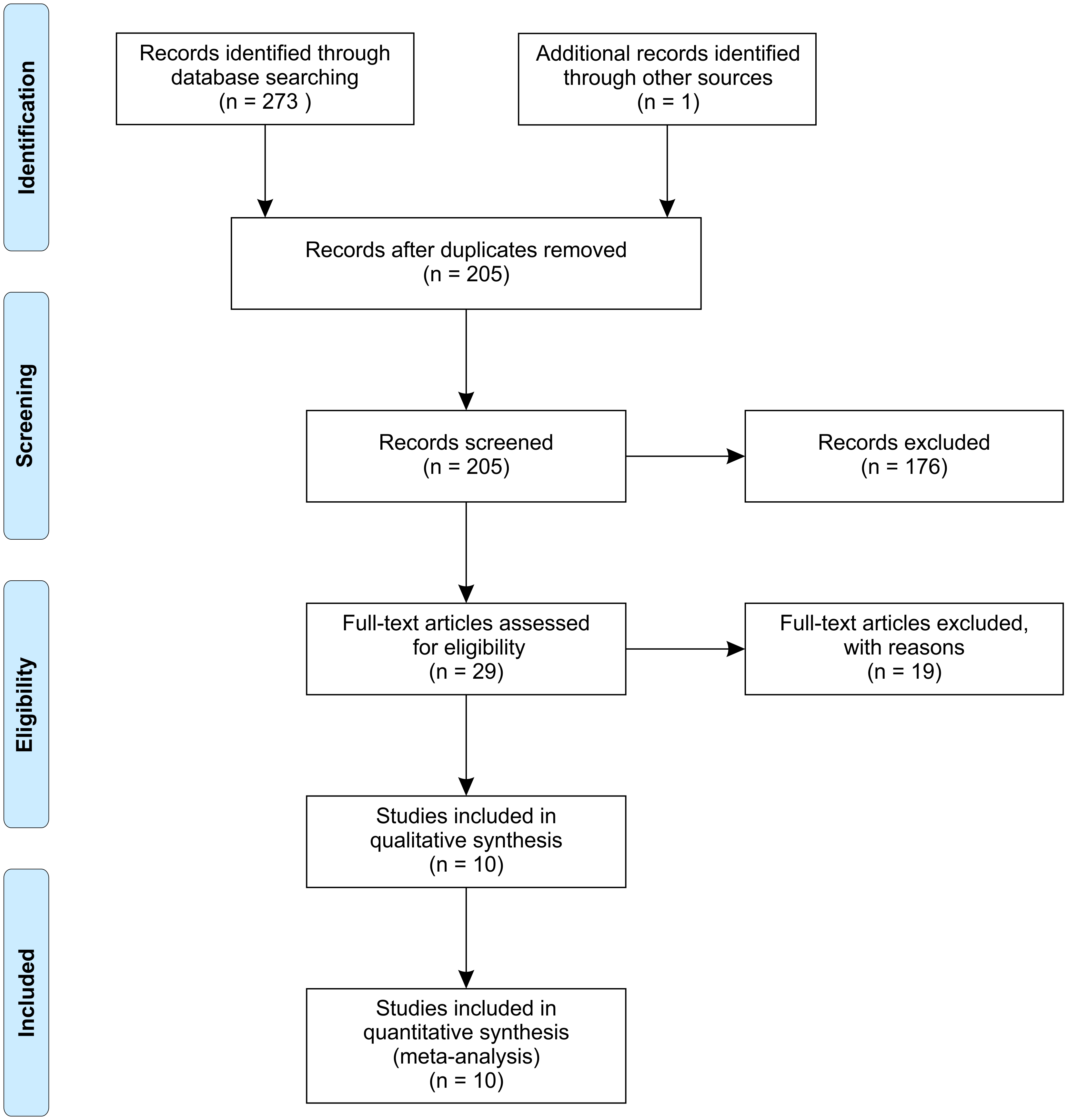

Supplement: S1 Fig — (TIF) [file pone.0221046.s002.tif]
